# Green sonochemical synthesis of silver nanoparticles at varying concentrations of κ-carrageenan

**DOI:** 10.1186/s11671-015-0916-1

**Published:** 2015-07-28

**Authors:** Randa Fawzi Elsupikhe, Kamyar Shameli, Mansor B Ahmad, Nor Azowa Ibrahim, Norhazlin Zainudin

**Affiliations:** Department of Chemistry, Faculty of Science, Universiti Putra Malaysia, 43400 Serdang, Selangor Malaysia; Malaysia-Japan International Institute of Technology (MJIIT), Universiti Teknologi Malaysia, Jalan Sultan Yahya Petra (Jalan Semarak), 54100 Kuala Lumpur, Malaysia

**Keywords:** Green method, Ultrasonic irradiation, Silver nanoparticles, κ-carrageenan, Silver nitrate

## Abstract

A green sonochemical method was developed for preparing silver nanoparticles (Ag-NPs) in different concentrations of kappa carrageenan (κ-carrageenan). The κ-carrageenan was used as a natural eco-friendly stabilizer, and ultrasonic irradiation was used as a green reducing agent. The number of Ag-NPs increased with increasing κ-carrageenan concentrations. Formation of Ag/κ-carrageenan was determined by UV-visible spectroscopy where the surface plasmon absorption maximum was observed at 402 to 420 nm. The X-ray diffraction (XRD) analysis showed that the Ag-NPs are of a face-centered cubic structure. The Fourier transform infrared (FT-IR) spectrum indicated the presence of Ag-NPs in κ-carrageenan. Transmission electron microscopy (TEM) image for the highest concentration of κ-carrageenan showed the distribution of Ag-NPs with an average particle size near to 4.21 nm. Scan electron microscopy (SEM) images illustrated the spherical shape of the Ag-NPs. The use of photo irradiation provides a green and economic feature to this work.

## Background

In the past 10 years, researchers have paid great attention to the nanotechnology field, which deals with reaction at the atomic and molecular level. Nanotechnology contains the synthesis, characterization, and application of devices and materials whose smallest organization in at least one dimension is on a scale of less than 100 nm [1–3]. Nanotechnology offers a broad technological base for applications in several areas such as modeling, bioprocessing in industry, and molecular medicine [4–6].

Metallic nanoparticles are of interest especially in biomedical sciences and engineering because of their huge potential in nanotechnology, hence opening a wide range of potential applications in biotechnology and magnetic separations [7]. Metallic NPs also offer applications in biomedicine and drug delivery [8, 9].

Due to its good conductivity, catalytic properties, chemical stability, and antibacterial activity, silver nanoparticles have gained much interest [10, 11]. Silver nanoparticles (Ag-NPs) have different catalytic properties like surface plasmon resonance [12]. Ag-NPs possess strong toxicity against a wide range of microorganisms and bacterial cells and have long been used as potent bactericidal agents [13].

Generally, there are many methods for the synthesis of metal nanoparticles. They can be prepared by chemical and physical methods. The chemical method for the synthesis of Ag-NPs is by using a chemical reduction method [14, 15]. Physical methods for preparing metal nanoparticles as a green method can be done by using the irradiation as a reducing agent including gamma irradiation [16], UV-irradiation [17], microwave irradiation [18], and ultrasonic waves [19].

Sonochemistry is the research area in which molecules undergo a chemical reaction due to the application of powerful ultrasound radiation (20 KHz to 10 MHz) [20]. The sonochemical method has been studied for yielding different kinds of nanomaterials, especially noble metal nanoparticles, such as gold, platinum, and lead [21]. The sonofication mechanism is called cavitation which is the production of a radical species by generating bubbles in the solution. The bubbles grow in the solution, and when they reach maximum size, the bubbles collapse and generate high temperatures and pressure. These conditions cause breaking of chemical bonds and formation of free radicals.

Kappa carrageenan (κ-carrageenan) is found in numerous red seaweeds. This polysaccharide has a linear structure of sulfated polysaccharide of D-galactose and 3,6-anhydro-D-galactose [22]. In the food industry, κ-carrageenan is widely used for example as a gelling agent and as a texture improvement for cottage cheese. Also, it can be used in toothpaste; air freshener gels, cosmetic creams, and shoe polish [23]. The biological activity of carrageenan as a natural polysaccharide has carried a large increase in its use for human applications due to its chemical structure and physical properties, and κ-carrageenan has also been used in engineering for the preparation of drug vehicles for controlled release [24].

As κ-carrageenan is a cheap natural polymer that has a negative charge in its back bone, it can be used as a stabilizer for the synthesis of Ag-NPs. On the other hand, ultrasonic irradiation plays an important role as a reducing agent to form metal NPs with small size and high distribution. In the literature, there is no report on the synthesis of Ag-NPs by using κ-carrageenan as a stabilizer. Hence, in this work, we proposed a green synthesis method of Ag-NPs by reducing and varying the concentrations of κ-carrageenan under ultrasonic irradiation for 90 min at room temperature. For the preparation of Ag-NPs, the effect of κ-carrageenan concentration on the optical properties, structures, and morphologies of Ag-NPs were characterized by using ultraviolet-visible (UV-vis) spectroscopy, X-ray diffraction (XRD), Fourier transform infrared (FT-IR) spectroscopy, transmission electron microscopy (TEM), and scan electron microscopy (SEM). This is the first report in the literature on the synthesis of nanoparticles by using different concentrations of κ-carrageenan as a stabilizer with ultrasonic irradiation as a reducing agent to form small size and highly distributed Ag-NPs.

## Methods

All the reagents in this work were used as received without any purification. The κ-carrageenan was obtained from Sigma (St. Louis, MO, USA), grad type (CAS 9000-07-1), and AgNO_3_ was obtained from Bendosen 99.89% (C0721-2284551). The aqueous solutions were prepared by using double distilled water.

### Synthesis of silver nanoparticles

The Ag-NPs were synthesized by reducing AgNO_3_ using ultrasonic waves in the presence of κ-carrageenan. Five suspensions were prepared, by adding 10 mL of 0.1 M AgNO_3_ to 40-mL κ-carrageenan. The κ-carrageenan solutions used were 0.1, 0.15, 0.20, 0.25, and 0.3 wt%, respectively. The solutions were stirred for 1 h to obtain AgNO_3_/κ-carrageenan. Then, the samples were exposed to high-intensity ultrasound irradiation under amplitude of 50% for 90 min at room temperature. Ultrasound irradiation was carried out with ultrasonic liquid processors (Hielscher ultrasound UP-400S, Teltow, Brandenburg, Germany, 50/100 Hz) immersed directly into the reaction solution. After that, the suspensions were centrifuged for 15 min and washed with double distilled water four times to remove the silver ion residue. The nanoparticles were precipitated then dried at 40°C under vacuum overnight to obtain the Ag-NPs.

### Characterization methods and instruments

The Ag/κ-carrageenan nanoparticles were characterized using UV-vis spectroscopy, XRD, FT-IR spectroscopy, TEM, and SEM. The UV-vis spectra were recorded over a range of 300 to 800 nm with the H.UV.1650 PC, SHIMADZU UV-vis spectrophotometer (SHIMADZU, Kyoto, Japan). The XRD patterns were carried out on a Philips X’pert (Cu Kα, Philips, Amsterdam, Netherlands) and were recorded at a scan speed of 2°/min. FT-IR spectra were recorded over the range of 500 to 4,000 cm^−1^ with a series 100 PerkinElmer FT-IR 1650 spectrophotometer (PerkinElmer, Waltham, MA, USA). TEM observations were carried out on a Hitachi H-7100 electron microscope (Hitachi, Chiyoda, Tokyo, Japan), and the particle size distributions were determined using the UTHSCSA Image Tool version 3.00 program. SEM was carried out on Jeol-JSM-7600F (Jeol, Tokyo, Japan).

## Results and discussion

**Figure 1:**
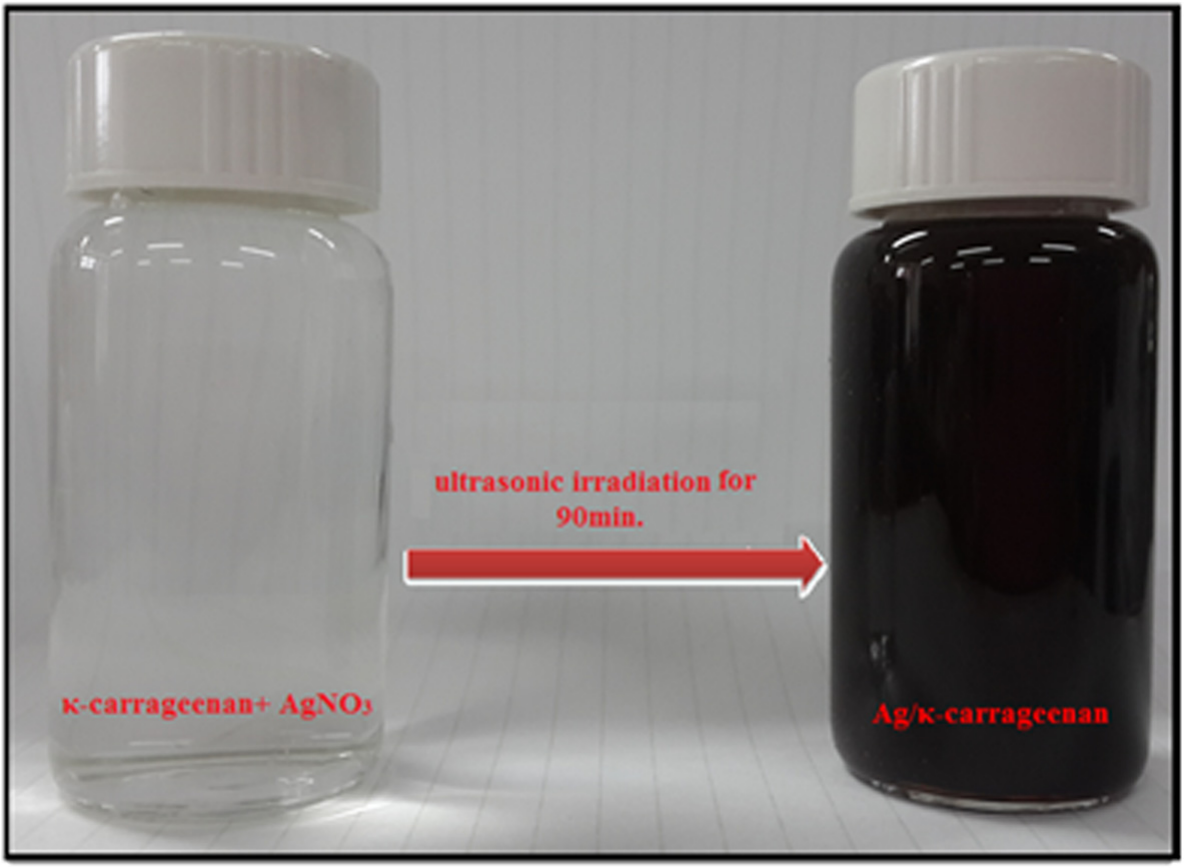
Photograph of Ag^+^/κ-carrageenan and Ag /κ-carrageenan.

The κ-carrageenan and AgNO_3_ was a colorless suspension; when the suspension was exposed to ultrasonic irradiation at amplitude 50% for 90 min at room temperature, the color changed from colorless to dark brown indicating the formation of the Ag-NPs in the κ-carrageenan suspensions shown in Figure 1.

The mechanism of formation of Ag-NPs is proposed by Equations 1, 2, 3, 4, 5 and 6. After application of ultrasonic waves in the AgNO_3_/κ-carrageenan aqueous suspensions, H radicals and OH free radicals formed as in Equation 1. The OH radicals reacted with the H atom in the κ-carrageenan group and formed free radicals in the polymer group [25], as shown in Equation 2

1 $$ {\mathrm{nH}}_2\mathrm{O}\overset{\ \mathrm{Sonicate}\ }{\to } + \mathrm{H}+\mathrm{O}\mathrm{H} $$

2 $$ \mathrm{O}\mathrm{H} + \mathrm{R}\mathrm{H}\to \mathrm{R}+{\mathrm{H}}_2\mathrm{O} $$

RH refers to the κ-carrageenan polymer. On the other hand, AgNO_3_ separated into Ag^+^ and ${\mathrm{NO}}_3^{-}$ ions in the aqueous solution [16] in Equation 3.

3 $$ {\mathrm{Ag}\mathrm{NO}}_3\overset{\mathrm{Hydrolysis}}{\to }{\mathrm{Ag}}^{+} + {\mathrm{NO}}_3^{-} $$

The free radical in Equation 2 reduced Ag^+^ to form Ag*°* and a new group R^'^) [26] in Equation 4.

4 $$ \mathrm{R} + {\mathrm{Ag}}^{+}\to {\mathrm{Ag}}^{{}^{\circ}}+{\mathrm{R}}^{\hbox{'}}+{\mathrm{H}}^{+} $$

Also, the H radical can reduce Ag^+^ to form Ag*°* [27] as seen in Equation 5.

5 $$ {\mathrm{Ag}}^{+}+\mathrm{H}\overset{\mathrm{reductions}}{\to }{\mathrm{Ag}}^{{}^{\circ}} $$

Equation 6 refers to the direct reaction of Ag^+^ with water

6 $$ {\mathrm{Ag}}^{+}+{\mathrm{H}}_2\mathrm{O}\to {\mathrm{Ag}}^{{}^{\circ}}+\mathrm{O}\mathrm{H} + {\mathrm{H}}^{+} $$

### UV-visible spectroscopic analysis

**Figure 2:**
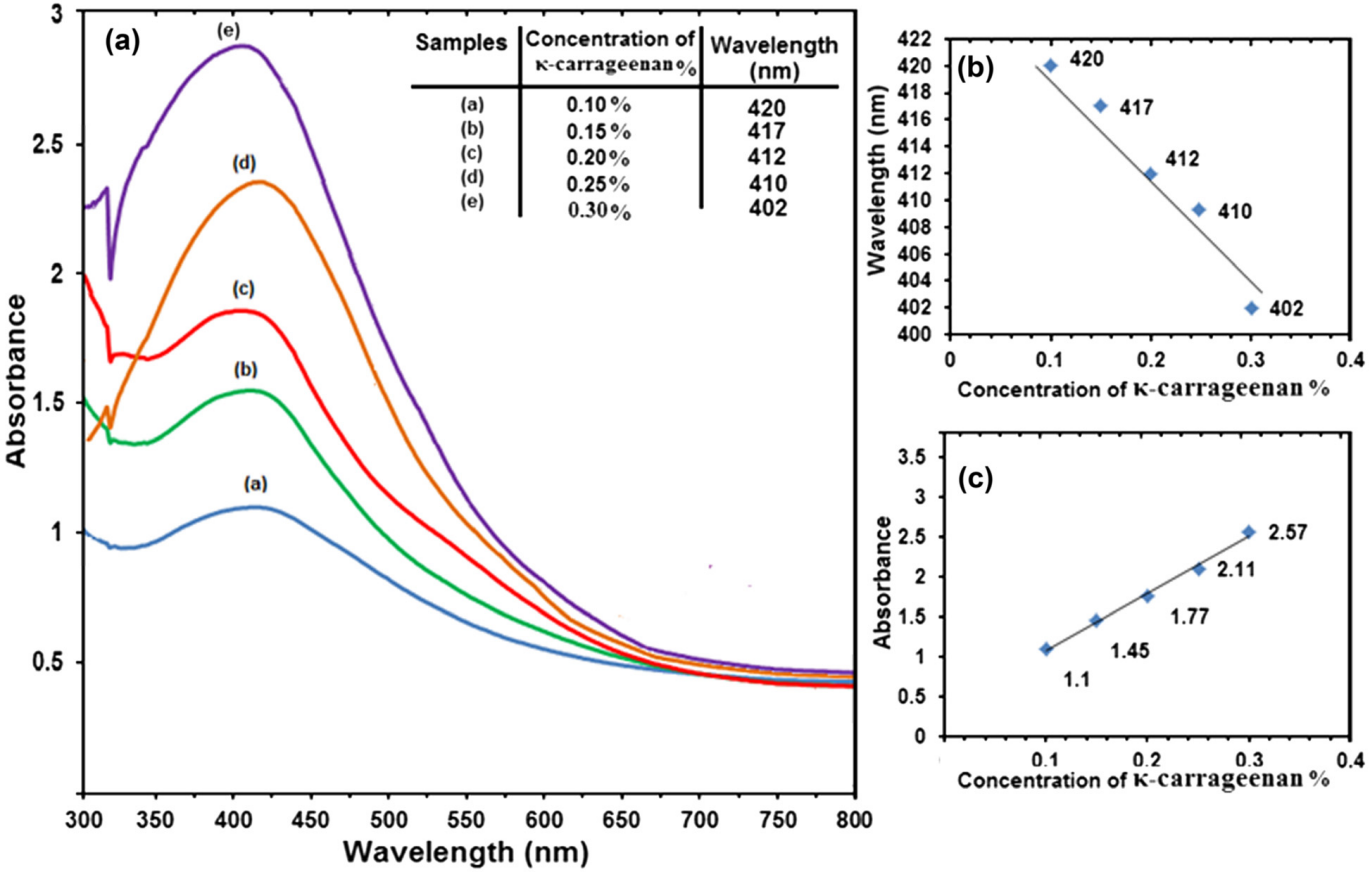
UV-visible absorption spectra for Ag/κ-carrageenan at different concentrations of κ-carrageenan (**a**, **b**, and **c**) for 0.1%, 0.15%, 0.2%, 0.25%, and 0.3%, respectively (a-e).

UV-visible spectroscopy data determined the formation of Ag-NPs by observing the surface plasmon resonance (SPR) bands. Figure 2a,b,c shows the preparation of Ag-NPs in κ-carrageenan by using different concentrations of κ-carrageenan. Commonly, the absorption spectrum of NP_S_ depends on the shape, size, and size distribution of the nanoparticles [28]. However, SPR band characteristic of Ag-NPs was identified around 402 to 420 nm (Figure 2a), which indicates the formation of Ag-NPs [29].

As shown in Figure 2a, when the concentrations of κ-carrageenan increased (0.1%, 0.15%, 0.2%, 0.25%, and 3% in a, b, c, d, and e, respectively), the intensity of the SPR peak also regularly increased. The increase of the absorbance was indicative that the concentration of Ag-NPs increased [30]

Furthermore, Figure 2b shows that with an increase of the concentrations of κ-carrageenan, the absorbance also increased and shifted to lower wavelength at 402 nm to blue-shift, which referred to a decrease in the particle size [31, 32]. Based on Mie’s theory [33], nanoparticles with different sizes should demonstrate different optical properties due to the difference in the SPR bands.

In Figure 2c it was observed that the 0.3% κ-carrageenan solution had a larger absorbance compared to other samples. The increase of the absorbance indicated that the concentration of Ag-NPs increased [31].

### X-ray diffraction analysis

**Figure 3:**
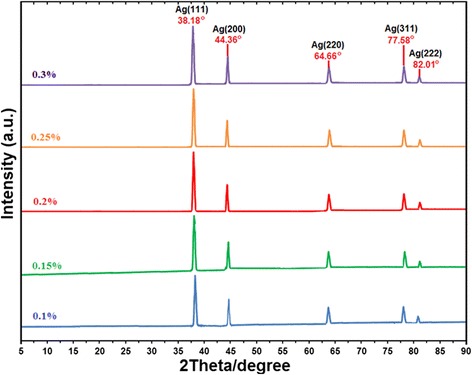
X-ray diffraction patterns for the Ag/κ-carrageenan at different concentrations of κ-carrageenan (0.1%, 0.15%, 0.2%, 0.25%, and 0.3%).

The X-ray diffraction (XRD) patterns of the prepared Ag/κ-carrageenan at different concentrations of κ-carrageenan indicated the formation of the Ag-NPs. As seen in Figure 3, all the samples had the same diffraction profiles. The XRD peaks at 2θ of 38.18°, 44.36°, 64.66°, 77.58°, and 82.01° can be attributed to the (111), (200), (220), (311), and (222) crystallographic planes of the face-centered cubic (fcc) silver crystals, respectively (Ref. # 01-087-0597). For all the samples, the main crystalline phase was silver with no obvious impurities [34, 35].

### FT-IR chemical analysis

**Figure 4:**
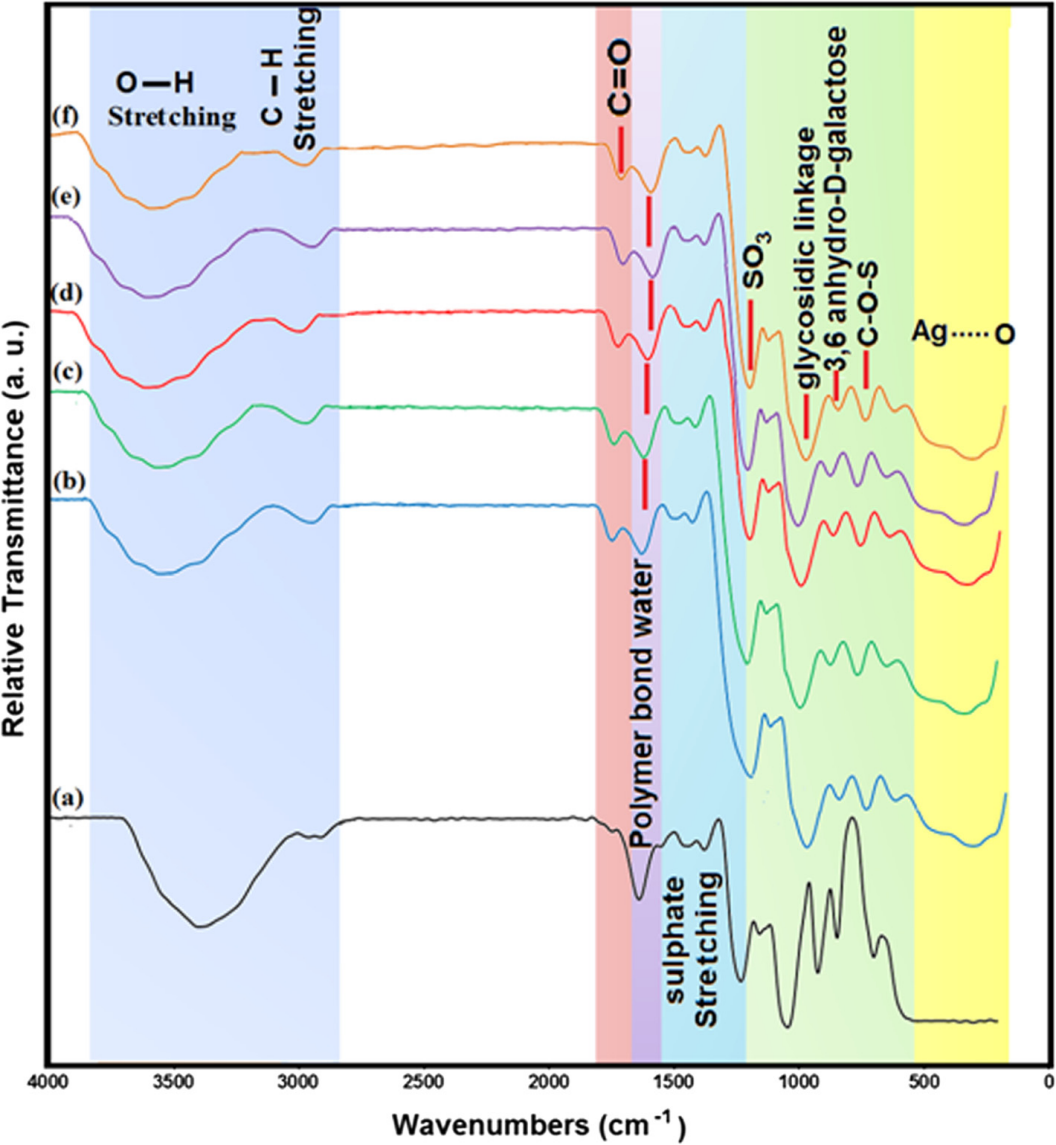
The FT-IR spectra for κ-carrageenan (**a**) and Ag/κ-carrageenan at different concentrations of κ-carrageenan [0.1%, 0.15%, 0.2%, 0.25%, and 0.3%, respectively (**b**-**f**)].

**Figure 5:**
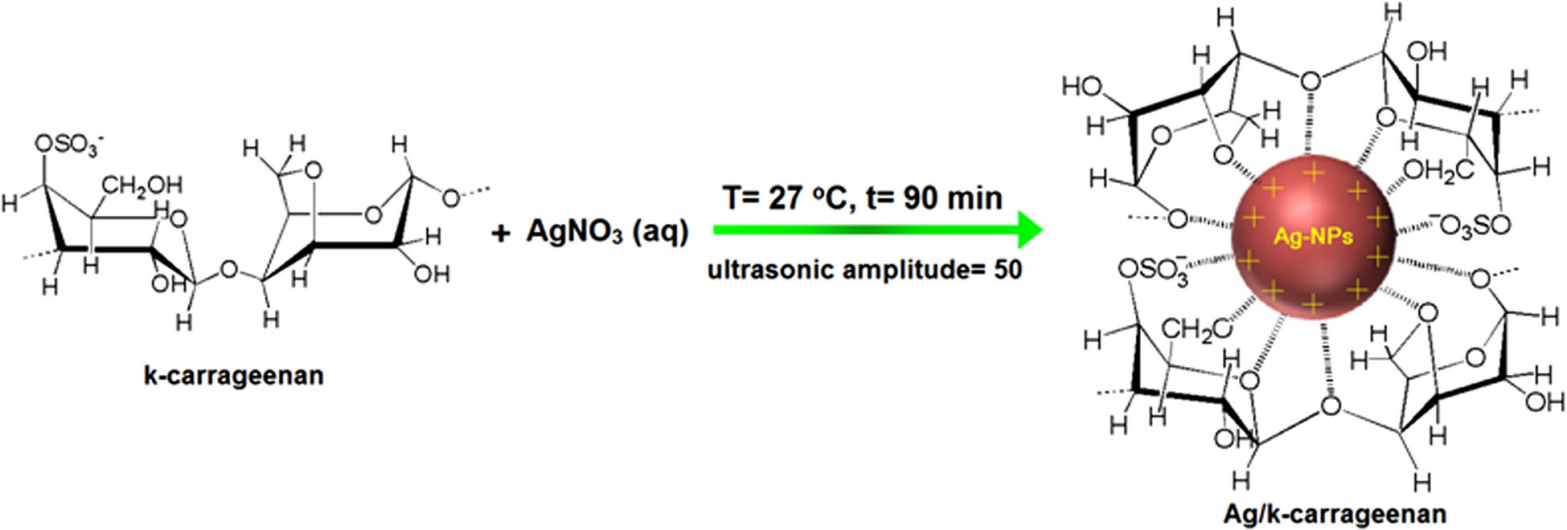
Schematic diagram illustrating the interaction between the Ag-NPs charged groups that are capped with κ-carrageenan.

FT-IR results confirmed the interactions of Ag-NPs obtained with κ-carrageenan. Figure 4 shows the FT-IR spectra of pure κ-carrageenan (a) and Ag/κ-carrageenan with different concentrations of κ-carrageenan (0.10%, 0.15%, 0.20%, 0.25%, and 0.3% (b-f)), respectively. In Figure 4(a), the absorption observed at 3,385 cm^−1^ was characteristic of the O-H stretching, absorption at 2,912 cm^−1^ was due to the interlayer C-H stretching, absorption at 1,636 cm^−1^ for polymer bond water, absorption at 1,446 cm^−1^ for sulfate stretch, absorption at 1,238 cm^−1^ for ester sulfate group C = O, absorption at 1,048 cm^−1^ for glycosidic linkage, absorption at 924 cm^−1^ for 3,6-anhydro-D-galactose, and absorption at 847 cm^−1^ for C-O-S axial secondary sulfate on C-4 of galactose [36, 37]. However, after adding AgNO_3_ and applying ultrasonic irradiation in Figure 4(b-f), a new peak at 1,757 cm^−1^ appeared, which was due to the formation of the carbonyl group. The carbonyl group resulted from the oxidation of carbohydrate radicals generated inside the carrageenan polymer [38]. The broad peak in the range 100 to 500 cm^−1^ was related to Ag-NPs banding with oxygen from hydroxyl groups of κ-carrageenan chains [35]. This is according to the presence of van der Waals forces between the positively charged groups that surround the surface of the inert Ag-NPs and negatively charged groups presenting in the molecular structure of the κ-carrageenan as shown in Figure 5. Moreover, the change in wavenumber in the Ag/κ-carrageenan samples that shifted to the lower wave numbers indicated interaction between κ-carrageenan and Ag-NPs [39–41].

### Morphology study

**Figure 6:**
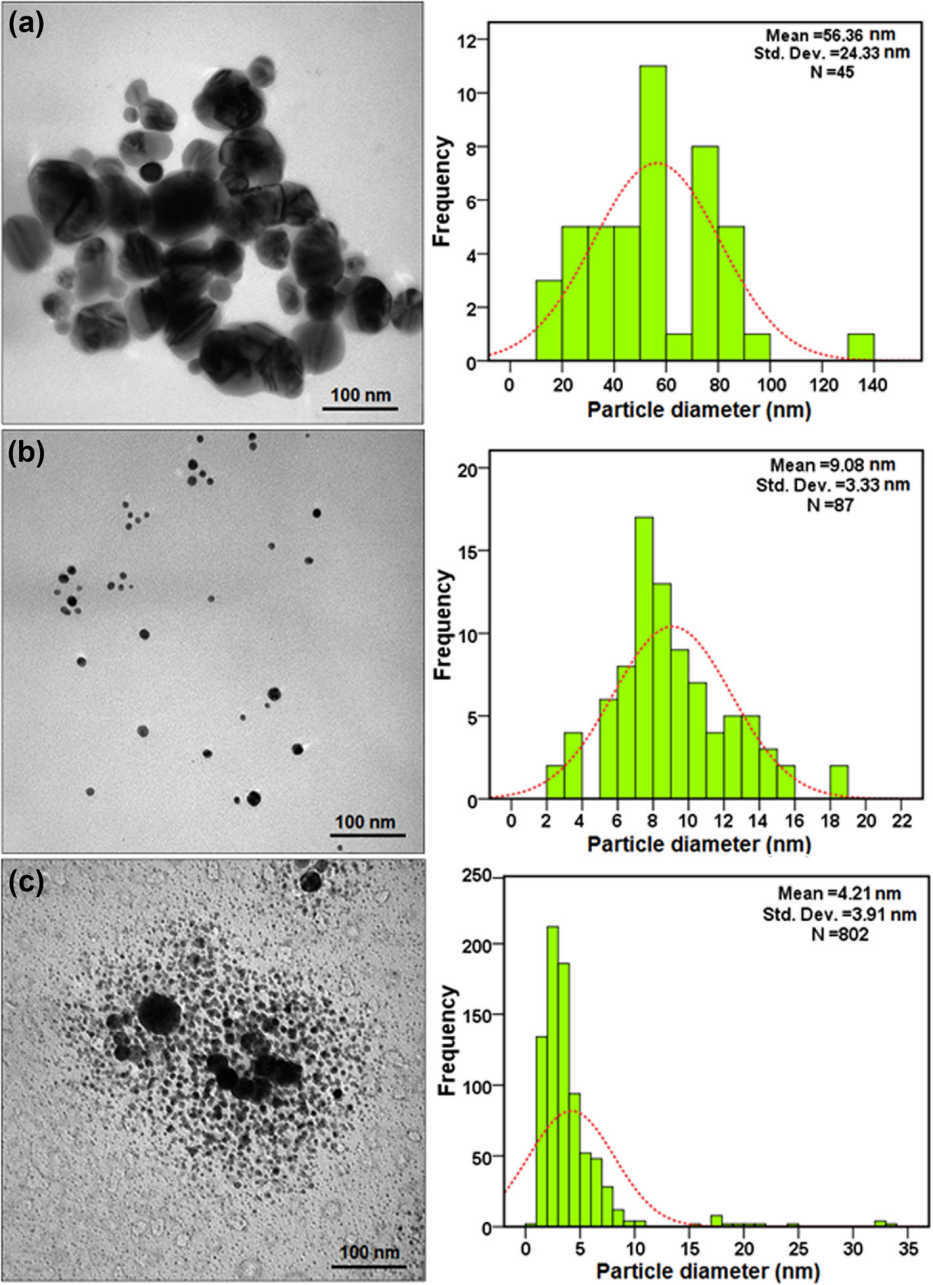
TEM images and corresponding size distributions for Ag/κ-carrageenan at different concentrations of κ-carrageenan. [0.1%, 0.2%, and 0.3%, respectively (**a**, **b**, **c**)].

The TEM images and their corresponding particle size distributions for Ag-NPs at different concentrations of κ-carrageenan are shown in Figure 6a, b, c for 0.1%, 0.2%, and 0.3%, respectively. TEM images and their size distributions indicated that the mean diameters and standard deviation of Ag-NPs were about 56.36 ± 24.33, 9.08 ± 3.33, and 4.21 ± 3.91 nm for 0.1%, 0.2%, and 0.3%, respectively. The numbers of Ag-NPs counted for TEM images were around 45, 87, and 802 for 0.1%, 0.2%, and 0.3%, respectively. The TEM results were in agreement with the UV-vis spectral data and indicated that when the concentrations of κ-carrageenan were increased, the size of Ag-NPs decreased with increasing distribution.

**Figure 7:**
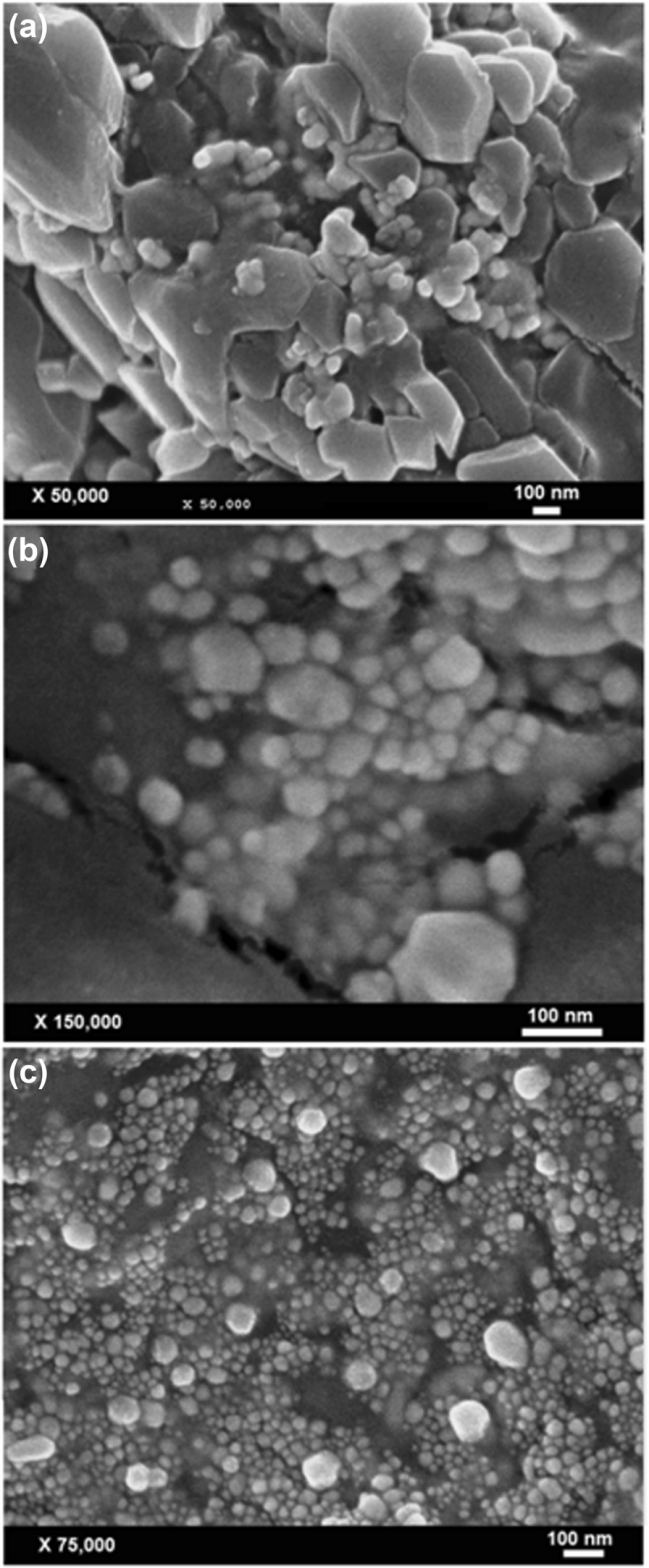
SEM images for Ag/κ-carrageenan at different concentrations of κ-carrageenan. [0.1%, 0.2%, and 0.3 %, respectively (**a**, **b**, **c**)].

SEM images for Ag-NPs at different concentrations of κ-carrageenan (0.1%, 0.2%, and 0.3%, respectively) are shown in Figure 7a,b,c. SEM images indicated the change in the surface of Ag/κ-carrageenan, when the concentration of κ-carrageenan increased. In Figure 7a, the formation of Ag-NPs with a bigger size was observed. However, Figure 7b shows the size of Ag-NPs became smaller with increasing concentration of κ-carrageenan. Moreover, Figure 7c shows the smallest size and numbers of Ag-NPs also increased, that referring to, the yielding of Ag-NPs increased with smaller spherical size when the concentration of κ-carrageenan increased. These results were in agreement with UV-vis and TEM data.

## Conclusions

In summary, we reported the successful use of ultrasonic irradiation as a green and physical reducing method for the synthesis of Ag-NPs in different concentrations of κ-carrageenan. The Ag-NPs were successfully formed as proven by the maximum surface plasmon resonance peak at 402 to 420 nm for each sample as indicated by UV-vis spectroscopy. The XRD pattern also showed the fcc crystal structure of Ag-NPs without any impurity for all the samples. FT-IR showed the interactions that exist between κ-carrageenan and Ag-NPs. The TEM images and their particle size distributions indicated that as κ-carrageenan concentrations increased, the number of Ag-NPs also increased. SEM data showed that with increasing κ-carrageenan concentration, there were change in the surface of Ag/κ-carrageenan, where small-sized Ag-NPs with spherical shape were obtained.

## References

[1] Roco MC (2003). Nanotechnology: convergence with modern biology and medicine. Curr Opin Biotechnol.

[2] Silva GA (2004). Introduction to nanotechnology and its applications to medicine. Surg Neurol.

[3] Shameli K, Ahmad MB, Al-Mulla EAJ, Shananzadeh P, Bagheri S. Antibacterial effect of silver nanoparticles on talc composites. Res Chem Intermed. 2013;1–13. doi: 10.1007/s11164-013-1188-y

[4] Shabanzadeh P, Yusof R, Shameli K. Artificial neural network for modeling the size of silver nanoparticles prepared in montmorillonite/starch bionanocomposites. J Ind Eng Chem. 2014;1–9. doi: 10.1016/j.jiec.2014.09.007

[5] Shabanzadeh P, Senu N, Shameli K, Ismail F, Zamanian A, Mohagheghtabar M. Prediction of silver nanoparticles’ diameter in montmorillonite/chitosan bionanocomposites by using artificial neural networks. Res Chem Intermed. 2013;1–13. doi: 10.1007/s11164-013-1431-6

[6] Jazayeri SD, Ideris A, Zakaria Z, Shameli K, Moeini H, Omar AR (2012). Cytotoxicity and immunological responses following oral vaccination of nanoencapsulated avian influenza virus H5 DNA vaccine with green synthesis silver nanoparticles. J Control Release.

[7] Mody VV, Siwale R, Singh A, Mody HR (2010). Introduction to metallic nanoparticles. J Pharm Bioallied Sci.

[8] Ahmad MB, Shameli K, Wan Yunus WMZ, Zargar M (2009). Synthesis and antibacterial activity of silver/montmorillonite nanocomposites. Res J Biol Sci.

[9] Kogan MJ, Olmedo I, Hosta L, Guerrero AR, Cruz LJ, Albericio F (2007). Peptides and metallic nanoparticles for biomedical applications. Nanomedicine.

[10] Ahmad MB, Shameli K, Wan Yunus WMZ, Zargar M (2009). Antibacterial activity of silver/clay/chitosan bionanocomposites. Res J Biol Sci.

[11] Satyavani K, Gurudeeban S, Ramanathan T, Balasubramanian T (2011). Biomedical potential of silver nanoparticles synthesized from calli cells of *Citrullus colocynthis* (L.) Schrad. J Nanobiotechnology.

[12] Ayala-Núñez NV, Villegas HHL, Turrent LDCI, Padilla CR (2009). Silver nanoparticles toxicity and bactericidal effect against methicillin-resistant *Staphylicoccus aureus*: nanoscale does matter. Nanobiotechnology.

[13] Abdeen S, Geo S, Sukanya S, Praseetha P, Dhanya R (2013). Biosynthesis of silver nanoparticles from actinomycetes for therapeutic applications. Int J Nano Dimens.

[14] Guzmán MG, Dille J, Godet S (2009). Synthesis of silver nanoparticles by chemical reduction method and their antibacterial activity. Int J Chem Biomol Eng.

[15] Shameli K, Ahmad MB, Wan Yunus WMZ, Jokar M (2010). Synthesis and characterization of silver/polylactide nanocomposites. Proc World Acad Sci Eng Technol..

[16] Shameli K, Ahmad MB, Wan Yunus WMZ, Gharayebi Y, Sedaghat S (2010). Synthesis of silver/montmorillonite nanocomposites using γ-irradiation. Int J Nanomed..

[17] Shameli K, Ahmad MB, WanYunus WMZ, Rustaiyan A, Zargar M, Abdullahi Y (2010). Green synthesis of silver/montmorillonite/chitosan bionanocopmosites using the UV irradiation method and evaluation of antibacterial activity. Int J Nanomed..

[18] Tsuji M, Hashimoto M, Nishizawa Y, Kubokawa M, Tsuji T (2005). Microwave assisted synthesis of metallic nanostructures in solution. Chem-Eur J.

[19] Elsupikhe RF, Shameli K, Ahmad MB. Effect of ultrasonic radiation’s times to the control size of silver nanoparticles in κ-carrageenan. Res Chem Intermediat, 2015:1-10. doi: 10.1007/s11164-015-1931-720.

[20] Gedanken A (2004). Using sonochemistry for the fabrication of nanomaterials. Ultrason Sonochem.

[21] He C, Liu L, Fang Z, Li J, Guo J, Wei J (2014). Formation and characterization of silver nanoparticles in aqueous solution via ultrasonic irradiation. Ultrason Sonochem.

[22] Necas J, Bartosikova L (2013). Carrageenan: a review. Vet Med-Czech.

[23] Li L, Ni R, Shao Y, Mao S (2014). Carrageenan and its applications in drug delivery. Carbohydr Poly..

[24] Prajapati VD, Maheriya PM, Jani GK, Solanki HK (2014). Carrageenan: a natural seaweed polysaccharide and its applications. Carbohydr Poly..

[25] Nagata Y, Watananabe Y, Fujita S, Dohmaru T, Taniguchi S (1992). Formation of colloidal silver in water by ultrasonic irradiation. J Chem Soc, Chem Commun..

[26] Relleve L, Nagasawa N, Luan L, Yagi T, Aranilla C, Abad L (2005). Degradation of carrageenan by radiation, polymer degradation and stability. Polym Degrad Stability.

[27] Chaodong H, Lanlan L, Zeguo F, Jia L, Jinbao G, Jie W (2014). Formation and characterization of silver nanoparticles in aqueous solution via ultrasonic irradiation. Ultrason Sonochem..

[28] Hebeish A, Hashem M, El-Hady M, Sharaf S (2013). Development of CMC hydrogels loaded with silver nano-particles for medical applications. Carbohydr Poly.

[29] Remita S, Fontaine P, Lacaze E, Borensztein Y, Sellame H, Farha R (2007). X-ray radiolysis induced formation of silver nano-particles: A SAXS and UV–visible absorption spectroscopy study. NuclInstr Meth Phys Res B.

[30] Huang H, Yang X (2004). Synthesis of polysaccharide-stabilized gold and silver nanoparticles: a green method. Carbohydr Res.

[31] Zargar M, Shameli K, Najafi GR, Farahani F (2014). Plant mediated green biosynthesis of silver nanoparticles using *Vitex negundo* L. extract. J Ind Eng Chem.

[32] Shameli K, Ahmad MB, Shabanzadeh P, Al-Mulla EAJ, Zamanian A, Abdollahi Y (2014). Effect of *Curcuma longa* tuber powder extract on size of silver nanoparticles prepared by green method. Res Chem Intermed..

[33] Heath J (1989). Size-dependent surface-plasmon resonances of bare silver particles. Phys Rev B: Condens Matter.

[34] Krstić J, Spasojević J, Radosavljević A, Šiljegovć M, Kačarević-Popović Z (2014). Optical and structural properties of radiolytically in situ synthesized silver nanoparticles stabilized by chitosan/poly (vinyl alcohol) blends. Radiat Phys Chem..

[35] Shameli K, Ahmad MB, Jazayeri SD, Shabanzadeh P, Sangpour P, Jahangirian H (2012). Investigation of antibacterial properties silver nanoparticles prepared via green method. Chem Cent J.

[36] Pereira L, Amado AM, Critchley AT, Van de Velde F, Ribeiro-Claro PJ (2009). Identification of selected seaweed polysaccharides (phycocolloids) by vibrational spectroscopy (FTIR-ATR and FT-raman). Food Hydrocolloids.

[37] Pourjavadi A, Harzandi A, Hosseinzadeh H (2004). Modified carrageenan 3. Synthesis of a novel polysaccharide-based superabsorbent hydrogel via graft copolymerization of acrylic acid onto kappa-carrageenan in air. Eur Poly J.

[38] Abad L, Kudo H, Saiki S, Nagasawa N, Tamada M, Katsumura Y (2009). Radiation degradation studies of carrageenans. Carbohydr Poly.

[39] Shameli K, Ahmad MB, Jazayeri SD, Sedaghat S, Shabanzadeh P, Jahangirian H (2012). Synthesis and characterization of polyethylene glycol mediated silver nanoparticles by the green method. Int J Mol Sci.

[40] Li XY, Liu B, Ye WJ, Wang XY, Sun RC (2015). Effect of rectorite on the synthesis of Ag NP and its catalytic activity. Mater Chem Phys..

[41] Luo YQ, Shen SQ, Luo J, Wang XY, Sun RC (2015). Green synthesis of silver nanoparticle in xylan solution via Tollens reaction and its detection for Hg^2+^. Nanoscale..

